# Systematic history and updated generic key of the tribe Spathicarpeae (Aroideae, Araceae)

**DOI:** 10.3897/phytokeys.269.171742

**Published:** 2026-01-20

**Authors:** Elmar J. Hentz Júnior, Livia G. Temponi, Marcus A. Nadruz Coelho, José Fernando A. Baumgratz

**Affiliations:** 1 Escola Nacional de Botânica Tropical, Instituto de Pesquisas do Jardim Botânico do Rio de Janeiro, Rua Pacheco Leão, 2040, Rio de Janeiro, Brazil Escola Nacional de Botânica Tropical, Instituto de Pesquisas do Jardim Botânico do Rio de Janeiro Rio de Janeiro Brazil; 2 Herbário UNOP, Universidade Estadual do Oeste do Paraná, Rua Universitária, 1619, Cascavel, Paraná, Brazil Universidade Estadual do Oeste do Paraná Cascavel Brazil; 3 Instituto de Pesquisas do Jardim Botânico do Rio de Janeiro, Diretoria de Pesquisas, Rua Pacheco Leão, 915, Rio de Janeiro, Brazil Instituto de Pesquisas do Jardim Botânico do Rio de Janeiro Rio de Janeiro Brazil

**Keywords:** historical taxonomy, Neotropics, systematic botany

## Abstract

The Neotropical tribe Spathicarpeae (Araceae, Aroideae) comprises 14 genera distributed from southern Mexico to Uruguay. Despite its complex taxonomic history, the tribe lacks a comprehensive historical synthesis that could support future phylogenetic and taxonomic studies. Herein we provide a historical review of the classification of Spathicarpeae based on detailed analysis of the pertinent literature, as well as the examination of specimens preserved in many herbaria. A generic identification key and taxonomic notes on various taxa are also presented. This study aims to serve as a framework for forthcoming phylogenetic analyses and taxonomic revisions within the tribe.

## Introduction

The Neotropical tribe Spathicarpeae Schott (Araceae Juss., Aroideae Arn.) is a monophyletic group comprising 14 genera (*Asterostigma* Fisch. & C.A.Mey, *Bognera* Mayo & Nicolson, *Croatiella* E.G.Gonç., *Dieffenbachia* Schott, *Gearum* N.E.Br., *Gorgonidium* Schott, *Incarum* E.G.Gonç., *Lorenzia* E.G.Gonç., *Mangonia* Schott, *Spathantheum* Schott, *Spathicarpa* Hook., *Synandrospadix* Engl., *Taccarum* Brongn., *Vivaria* O. Cabrera, Tinitana, Cumbicus, Prina & Herrera) which exhibit a geophytic habit, stamens fused into a synandrium, and 17 chromosomes (2n = 34) (see e.g., Cusimano et al. 2011; Haigh et al. 2022). Most members of this tribe occur exclusively in South America, and only *Dieffenbachia* extends through Central America and Mexico (Gonçalves et al. 2007).

The tribe shows great morphological variability, which, combined with molecular studies, have been used for its classification (Mayo et al. 1997; Gonçalves 2002; Gonçalves et al. 2007). However, despite the taxonomic complexity of this group, there is no published report on the history of its classification, with early insights presented only in a thesis by Gonçalves (2002). Moreover, despite the description of more recently established genera [i.e., *Incarum* and *Croatiella* E.G.Gonç. (Gonçalves 2005), *Lorenzia* (Gonçalves 2012), and *Vivaria* O.Cabrera, Tinitana, Cumbicus, Prina & Herrera (Cabrera et al. 2022)], there is no identification key of the tribe that includes all genera.

We aim to establish the taxonomic framework upon which a forthcoming phylogenetic analysis of the tribe Spathicarpeae will be based. We provide here a historical survey of the classification of the tribe, emphasizing works that contributed most to the circumscription of its genera. A synopsis of the 14 currently recognized genera, including their diagnostic characters, distribution, an identification key to the genera, and a set of photographic plates and drawings that highlight the morphological features most relevant to their recognition are included herein. This will allow a clear taxonomic reference for future phylogenetic hypotheses and facilitate consistent interpretation of generic boundaries.

## Material and methods

This work is based on analysis of the protologues of the first species described for each of the genera currently recognized in the tribe. All names related to the genera were checked on botanical indexing platforms [IPNI, POWO, and Tropicos] to find the bibliography necessary for such a study. Most of the bibliography was found online, especially in the BHL. Type specimens were examined directly, during visits to herbaria, or through high-resolution digital images made available by the institutions in virtual repositories. The analysis of the protologues considered the diagnostic criteria used in the original descriptions, as well as the circumscription used by the author to categorize the specimen in the tribe, with the taxonomical knowledge at that point in time. Furthemore, other manuscripts related to the classification and description of the family Araceae were also analyzed.

In addition to literature, specimens deposited in several herbaria were consulted to support the taxonomic synopsis and verify morphological characteristics relevant to the delimitation of the taxa. Specifically, the following herbaria were consulted: B, BM, BR, CTES, CORD, CVRD, FCQ, FURB, G, HAMAB, HAS, HCF, HUEFS, HUTPL, INPA, K, M, MBM, MO, NY, P, R, RB, RFFP, SPF, UB, UFMT, UNOP, UPCB, W, and WU (codes according to Thiers 2025 [continuously updated]).

**Figure 1.**
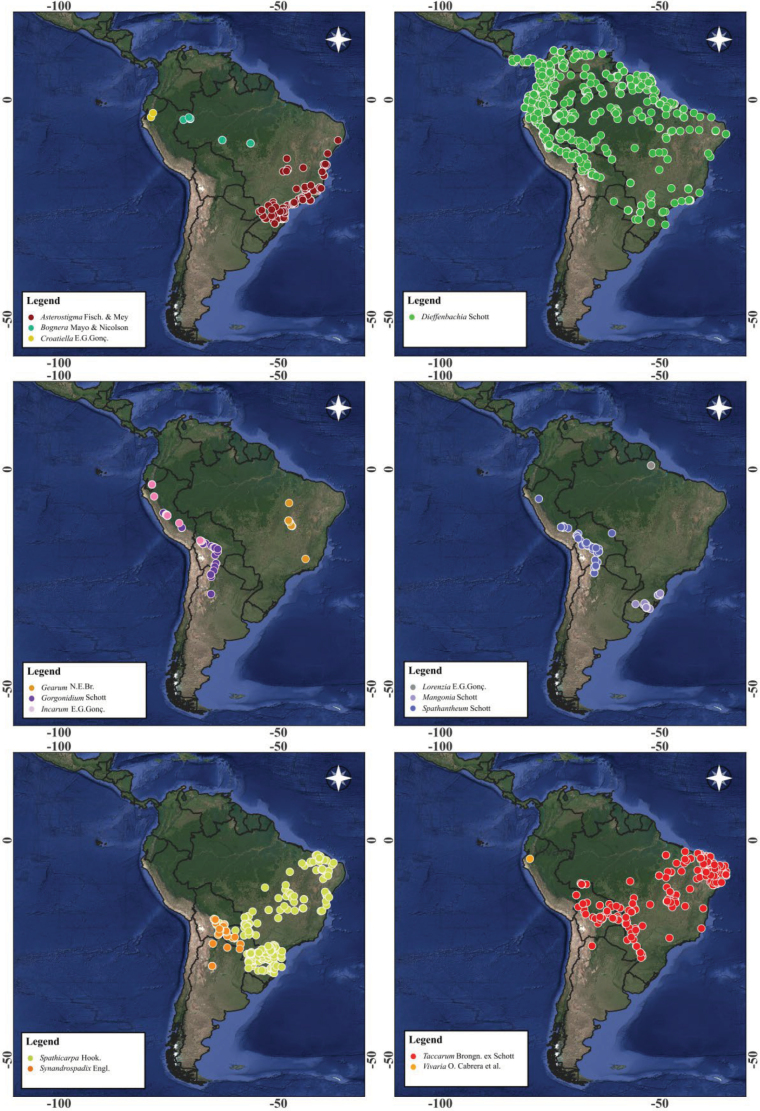
Geographic distribution of the genera of Spathicarpeae.

The botanical illustrations were produced manually, using ink, aiming to represent the morphological diversity among the genera of the tribe. The geographic distribution maps were prepared using QGIS software, based on the plotting of the databases SpeciesLink and GBIF containing georeferenced occurrences of each genus (Fig. 1). These data were previously organized and processed in RStudio, with additional manual corrections to ensure greater accuracy of the locations and to eliminate duplications.

## Results

### History of the tribe

The history of the classification of the tribe Spathicarpeae spans nearly 200 years, during which different circumscriptions have been proposed based on vegetative and floral characteristics, and more recently, on molecular data (Table 1).

**Table 1.** Description of the genera of Spathicarpeae in chronological order and method of inclusion in the tribe. M = Morphology, Mol = Molecular.

**Year**	**Genus**	**Method of inclusion**
1829	* Dieffenbachia *	First genera described
1831	* Spathicarpa *	(M): Spadix–spathe fusion
1845	* Asterostigma *	(M): Spadix–spathe fusion
1849	* Staurostigma *	*= Asterostigma*
1850	* Andromycia *	(M): Mixed material: *Xanthosoma* and *Asterostigma*
1857	* Mangonia *	(M): Spadix–spathe fusion
1857	* Taccarum *	(M): Spadix–spathe fusion
1859	* Spathantheum *	(M): Spadix completely fused to spathe
1859	* Rhopalostigmium *	= *Asterostigma*.
1864	* Gorgonidium *	(M): Spadix–spathe fusion
1882	* Gearum *	(M): Spadix–spathe fusion
1883	* Synandrospadix *	(M): Spadix–spathe fusion
1984	* Bognera *	(M): Spadix–spathe fusion
2005	* Incarum *	(M + Mol): Phylogenetically supported
2005	* Croatiella *	(M + Mol): Phylogenetically supported
2012	* Lorenzia *	(M + Mol): Phylogenetically supported
2022	* Vivaria *	(M + Mol): Phylogenetically supported

*Dieffenbachia* (Schott 1829: 803) was the first genus described for this tribe, when *D. seguine* (Jacq.) Schott was accepted based on *Arum seguine* Jacq. Later, Hooker (1831) described a new genus, *Spathicarpa*, establishing *S. hastifolia* based on a specimen collected by Jacobus Baird on the banks of the Uruguay River in Argentina. This circumscription was based on the observation that the material lacked a spadix, with flowers arranged in a line along the adaxial surface of the spathe, a feature previously unseen in the Araceae family.

In one of the first classifications for Araceae, Schott (1832: 20) proposed the tribe Anaporeae Schott, with two subtribes: (i) Spathicarpeae Schott, composed of the genera *Dieffenbachia* and *Spathicarpa*; (ii) Richardieae Schott, with *Aglaonema* Schott (currently placed in the tribe Aglaonemateae), *Homalonema* Schott (currently placed in the “philodendron clade”), and *Richardia* Kunth (synonym of *Zantedeschia* Spreng.) (Haigh et al. 2022). The subtribe Spathicarpeae was then characterized by the persistent spathe, a female zone of the spadix with lax flowers fused with the spathe, the unilocular ovary with a single ovule, with an erect, basifixed ovule and a capitate stigma, and a male zone with flowers having connate anthers and a truncate-peltate connective.

Later, Fischer and Meyer (1845: 148) described the genus *Asterostigma* based on a specimen originally collected in the province of Mato Grosso (Brazil), which was later cultivated at the Imperial Botanical Garden of Saint Petersburg. The authors emphasized that the genus did not present a unilocular ovary but rather a trilocular to tetralocular ovary. Nevertheless, the authors decided that it should be included in the tribe Anaporeae, subtribe Spathicarpeae, due to its resemblance to the group, mainly because of the partial fusion of the spadix with the spathe. They also argued that the subtribe Richardieae included species with unilocular to multilocular ovaries, so the number of locules should not be considered as a constant character for the circumscription of these subtribes.

Between 1845 and 1850, two other genera important to the history of the tribe were described: *Staurostigma* Scheidweiler (1849: 129), recorded from the province of São Paulo, Brazil; and *Andromycia* Richard (1850: 282) (both currently accepted as synonyms of *Asterostigma*), recorded from Cuba, in the surroundings of Havana.

With the advancement of morphological studies within the family, the tribe Spathicarpeae was proposed (Schott 1856) comprising the genera: *Asterostigma* (already including *Staurostigma* as its synonym), *Dieffenbachia*, and *Spathicarpa*. The author continued to highlight, as the main characteristics of this group, the spadix completely fused or fused only at the base to the spathe, uniovulate ovary locules (erroneously indicating this character for the tribe, since *Asterostigma* was already recognized at that time as having 3–4 ovules per locule), basifixed ovules, and anthers fused into synandria.

During this period, *Dieffenbachia* was already shown to be the richest genus of the tribe, with 15 species, while *Spathicarpa* remained monotypic (*S. hastifolia*), and *Asterostigma* included three species, *A. concinnum* Schott, *A. vellozianum* Schott, and *A. luschnathianum* Schott. *Andromycia* was not included in this classification.

Later, two new genera, *Mangonia* Schott (1857: 77) and *Taccarum* Brongn. ex Schott (1857: 221), were described. *Mangonia* was described based on a specimen that flowered in the private garden of J. D. Hooker, sent by Tweedie, who had collected the specimen in Uruguay. *Taccarum* was accepted after Schott analyzed the specimen of Ludwig Riedel s.n., collected in Brazil, in the province of Mato Grosso in 1826, and deposited at that time in the Herbarium of the Imperial Academy of Saint Petersburg, confirming that this specimen fully corresponded to the description previously communicated to him by Adolphe Théodore de Brongniart. In this publication, both genera were cited as belonging to the tribe Spathicarpeae, expanding its composition to five genera (*Asterostigma*, *Dieffenbachia*, *Mangonia*, *Spathicarpa*, and *Taccarum*).

Continuing his studies on Araceae, Schott described two new genera: *Spathantheum* Schott (1859a: 164), based on material collected in Bolivia by Alcide d’Orbigny, characterized by the spadix completely fused with the spathe, with male flowers in the apical portion and female flowers in the basal portion; and *Rhopalostigmium* Schott (1859b: 39) (currently *Asterostigma*), to accommodate a species collected in Ilhéus, Brazil, by Ludwig Riedel, distinguished by its trilobed stigma.

One year later, Schott (1860) proposed a new classification that included all the genera then subordinated to Spathicarpeae, separating them into two tribes: (i) Tribe Asterostigmeae, characterized by a spadix with female flowers at the base and male flowers at the apex, and anatropous ovules; (ii) Tribe Spathicarpeae, characterized by a spadix completely fused with the spathe, with female flowers on the outer part and male flowers on the inner part, and orthotropous ovules. Additionally, in this new circumscription, the tribe Asterostigmeae was divided into the following subtribes: Dieffenbachininae, characterized by having an aerial stem and leafy shoots, included only the genus *Dieffenbachia*; and Asterostigmatinae, characterized by having a tuberous stem and leaves and flowers that may emerge non-simultaneously, included the genera *Andromycia* A. Rich. (currently *Asterostigma*), *Asterostigma*, *Mangonia*, *Rhopalostigmium* Schott (currently *Asterostigma*), and *Taccarum*. Meanwhile, the tribe Spathicarpeae included only two genera: *Spathicarpa* and *Spathantheum*.

A few years after Schott (1860), the genus *Gorgonidium* was described (Schott 1864: 282), and it is characterized by filiform staminodes and free stamens with very elongated filaments. In its description, this genus was treated as belonging to a distinct tribe (tribe Gorgonidieae).

Almost 20 years after Schott’s classification, Adolf Engler, a recently hired taxonomist in the Munich Botanical Garden, was invited by Alphonse Louis Pierre Pyrame de Candolle to prepare a monograph for the family. In 1878 he summarized the Brazilian taxa in *Flora Brasiliensis*, and one year later published the full monograph in the *Monographiae Phanerogamarum* (1879). In his early works, new modifications were proposed for this group of plants. Engler placed *Dieffenbachia* in what he called the suborder Aglaonemoideae Engl., tribe Dieffenbachieae Schott, indicated by having a “stem resembling a trunk”. He also proposed the suborder Staurostigmoideae Engl., represented in Brazil by *Staurostigma* (currently *Asterostigma*), *Mangonia*, and *Taccarum*, grouped by having anatropous ovules. The tribe Spathicarpeae was positioned within the suborder Aroideae, represented in Brazil only by *Spathicarpa*, and two non-Brazilian genera, *Gorgonidium* and *Spathantheum*.

Two other genera were described after this classification: *Gearum*, described from a specimen found in the plains at low altitude in the state of Goiás, characterized by having pedate leaves (Brown 1882: 196); and *Synandrospadix*, previously considered *Asterostigma vermitoxicus* Griseb., a species occurring in Argentina, characterized by having sagittate-cordate leaves and stamens with anthers fused at the apex (Engler 1883: 61).

The culmination of Engler’s work was the publication of *Das Pflanzenreich* from 1905 to 1920. In the last volume, a major revision for the subfamilies Aroideae and Pistioideae was published. Most of the genera treated until then belonged to the subfamily Aroideae, only *Dieffenbachia* treated in the subfamily Philodendroideae. In this study, the tribe Spathicarpeae was renamed tribe Asterostigmateae Schott emend. Engl., including the genera: *Asterostigma*, *Andromycia*, *Gearum*, *Gorgonidium*, *Mangonia*, *Spathicarpa*, *Spathantheum*, *Synandrospadix*, and *Taccarum*.

More than 40 years later, Bogner (1969) recognized that *Andromycia* was, in part, *Asterostigma*, as he observed that this genus was described based on a specimen with mixed structures from distinct taxa, with a leaf of *Xanthosoma cubense* (Schott) Schott and an inflorescence of *Asterostigma*, a strictly South American genera.

Later, a new genus of Araceae was described from material originating in western Amazonas (Rio Javarí region), based on a plant originally placed in *Ulearum* Engl. by Madison. It was then named *Bognera* Mayo & Nicolson and noted as morphologically similar to *Dieffenbachia* and *Anubias* Schott. In that study, this genus was considered to belong to a separate tribe, the tribe Bognereae Nicolson (Nicolson 1984: 690).

Engler’s classification remained unchanged for a long time, until M. H. Grayum made a new classification covering all the taxa of Araceae based on his work with pollen biology. On this, the tribe reverted to the name Spathicarpeae by the principle of priority, being integrated into the subfamily Philodendroideae. It is worth noting that besides the inclusion of Spathicarpeae in this subfamily, this tribe was also placed in a group called the “Aglaonema Alliance,” which also included the tribes Dieffenbachieae and Bognereae, showing the proximity of these taxa, mainly due to their elongated and epigeal stems (Grayum 1984; Grayum 1990).

More recently, in the classification of Bogner & Nicolson for Araceae, the tribe was again positioned in the subfamily Aroideae, but the genus *Dieffenbachia* remained in the subfamily Philodendroideae, tribe Dieffenbachieae, while *Bognera* was placed in the tribe Anubiadeae. This can be recognized as the last classification of Araceae following the Engler system, which radically changed with the use of molecular analysis (Bogner and Nicolson 1991).

In the early 1980s J. C. French carried out substantial anatomical research on Araceae, which led him to doubt the previous classification from Engler. He later published the first molecular cladistic analysis of the family (French 1995) which broke with the subfamily structure of Engler and recognized instead a very large subfamily Aroideae consisting of all the unisexual flowered genera. As a result, tribes became the main grouping taxa within this large concept of the subfamily Aroideae. In this, Spathicarpeae was initially positioned within an informal grouping called “philodendroid”, suggesting a close relationship of this tribe with the genera *Dieffenbachia* and *Bognera*. However, in the work The Genera of Araceae (Mayo et al. 1997), these genera were again reallocated into two distinct tribes, grouping *Dieffenbachia* and *Bognera* in the tribe Dieffenbachieae. Notably, Keating (2000) reincluded *Dieffenbachia* and *Bognera* in the tribe Spathicarpeae, based exclusively on the molecular data from French and collaborators, although without providing detailed arguments for this reclassification.

In the early 2000s, Eduardo Gonçalves focused on this tribe for his PhD thesis (2002). His work was essential for the understanding of this group, with information from many collections and cultivated specimens, presenting a taxonomic synopsis for most of the genera of the tribe, many considered as preliminar and never published. He also provided a good base for his next studies, especially for the first thorough phylogeny of the tribe.

With the advancement of molecular phylogeny, further changes were necessary regarding the genera of the tribe Spathicarpeae, the first being the description of two new genera, *Incarum* and *Croatiella* (both Gonçalves 2005), from the recombination of the species *Asterostigma pavonii* Schott [= *Incarum pavonii* (Schott) E.G.Gonç.] and *Asterostigma integrifolium* Madison [= *Croatiella integrifolia* (Madison) E.G.Gonç.]. Both species presented orthotropic ovules, a characteristic that does not match the description of the genus *Asterostigma*, which led the author to highlight the need for a new phylogenetic analysis for the tribe. This is due to the fact that the only previous phylogenetic study that addressed this group with a significant number of species was that by French et al. (1995), which did not use morphological characters for analysis.

Subsequently, Gonçalves et al. (2007) published a new phylogenetic analysis for the tribe Spathicarpeae, combining plastid DNA markers *matK* and *trnL-F* and phenotypic data. In this work, the nesting of *Dieffenbachia* and *Bognera* within the tribe became evident, and the molecular data combined with a morphological matrix supported the segregation of the two new genera proposed by Gonçalves (2005); *Incarum* and *Croatiella*. In this work, Gonçalves indicated the presence of three distinct subtribes, namely, Dieffenbachineae, Bognerineae, and Spathicarpineae. After Gonçalves’ studies, several whole-family molecular analyses were made for Araceae, confirming his interpretation of the tribe (Cabrera et al. 2008; Cusimano et al. 2011; Nauheimer et al. 2012; Henriquez et al. 2014).

More recently, two new genera were described for Spathicarpeae: *Lorenzia* E.G.Gonç., from Amapá and characterized by having a rhizomatous stem with many small cataphylls and trichomes on the venation (Gonçalves 2012), and *Vivaria* O. Cabrera, Tinitana, Cumbicus, Prina & Herrera, from southern Ecuador and characterized by having fused staminodes, which differs from the other genera of the tribe found in the country (Cabrera et al. 2022). In the description papers of both genera, phylogenetic analyses were performed using a morphological matrix and the *matK* marker. The tribe remained monophyletic with these inclusions; however, *Lorenzia*, although strongly supported within the tribe, showed only moderate support as a sister group to the remaining genera (BS = 82%), and *Vivaria* clustered in a clade with *Spathantheum*, *Gorgonidium*, and *Incarum* in the published cladogram (Cabrera et al. 2022).

Haigh et al. (2022) conducted a new phylogenetic study of the family using nuclear DNA and whole-genome sampling. Although the sampling did not include all genera of Spathicarpeae, the tribe remained monophyletic, but the relationships among genera showed low support.

Building upon this historical framework, Hentz Júnior et al. (in prep.) are now undertaking the first comprehensive phylogenetic analysis of Spathicarpeae after the early work of Eduardo Gonçalves. This includes all genera of the tribe and all known species of *Asterostigma*, as well as the use of new molecular methodologies to better understand the relation between the genera and the evolution of the group. The resulting phylogeny will also serve as the foundation for the taxonomic revision of *Asterostigma*.

### Key to the genera of Spathicarpeae

**Table:** 

1	Rhizomatous stem	**2**
–	Tuberous stem	**3**
2	Parallel-pinnate venation	*** Dieffenbachia ***
–	Reticulate venation	**4**
3	Spadix entirely adnate to the spathe	**5**
–	Spadix free or only adnate at the female zone	**6**
4	Plant with sagittate leaf base, veins with trichomes; pistillate flowers surrounded by staminodes	*** Lorenzia ***
–	Plant with obtuse leaf base, glabrous veins; pistillate flowers not surrounded by staminodes	*** Bognera ***
5	Ovary 6–8-locular, female flowers below, male flowers above	*** Spathantheum ***
–	Ovary 1-locular, male and female flowers mixed (two central rows of male flowers and two outer rows of female flowers)	*** Spathicarpa ***
6	Plants with entire leaves	**7**
–	Plants with pinnate, bipinnate, or pedate leaves	**9**
7	Apical sterile male zone present, locules 2-ovulate	*** Mangonia ***
–	Apical sterile male zone absent, locules 1-ovulate	**8**
8	Stamens not convergent, anthers rounded at apex; 5–6 locules; stigma 5–6-lobed	*** Croatiella ***
–	Stamens convergent, anthers tapering at apex; 4 locules; capitate stigma	*** Synandrospadix ***
9	Pedate leaves with 7–13 linear leaflets	*** Gearum ***
–	Leaves not pedate	**10**
10	Anatropous ovules (non-Andean distribution)	**11**
–	Orthotropous ovules (Andean distribution)	**12**
11	Bipinnatifid or sub-dracontoid leaf, elongated synandria, thecae with a vertical opening, capitate or lobed stigma	*** Taccarum ***
–	Pinnatifid leaf, short synandria, thecae with a horizontal opening, strongly lobed stigma	*** Asterostigma ***
12	Fused staminodes	*** Vivaria ***
–	Free staminodes	13
13	Filiform staminodes	*** Gorgonidium ***
–	Obpyramidal staminodes	*** Incarum ***

### Synopsis of the genera of Spathicarpeae

#### Asterostigma

Taxon classificationPlantaeAlismatalesAraceae

Fisch. & C.A.Mey, Bull. Cl. Phys. Math. Acad. Imp. Sci. Saint Pétersbourg, ser. 2, 3: 148 (1845). Type: Asterostigma langsdorffianum Fisch. & C.A.Mey.

A920B572-9611-5BE9-B222-318C6F4DC67B

Figs 2a, 3a

= Staurostigma Scheidweiler, Allg. Gartenzeitung 16: 129 (1848). Type: Staurostigma odorum Scheidweiler.
= Andromycia A. Richard, R. de la Sagra, Hist. Fis. Cuba 11: 282 (1850). Type: Andromycia cubensis A. Richard.
= Rhopalostigmium Schott, Oesterr. Bot. Zeitschr. 9: 39 (1859); [Rhopalostigma B.D. Jackson, Index Kew 2: 713 (1895), *orth*. var., *non*. R.A.Philippi (1858)]. Type: Rhopalostigmium riedelianum Schott.

##### Diagnosis.

The species of *Asterostigma* can be recognized by their tuberous stems, pinnate leaves (not pedate), spadix free or with only part of the female zone adnate to the spathe, short synandria, thecae with horizontal dehiscence, strongly lobed stigmas, and anatropous ovules.

**Figure 2.**
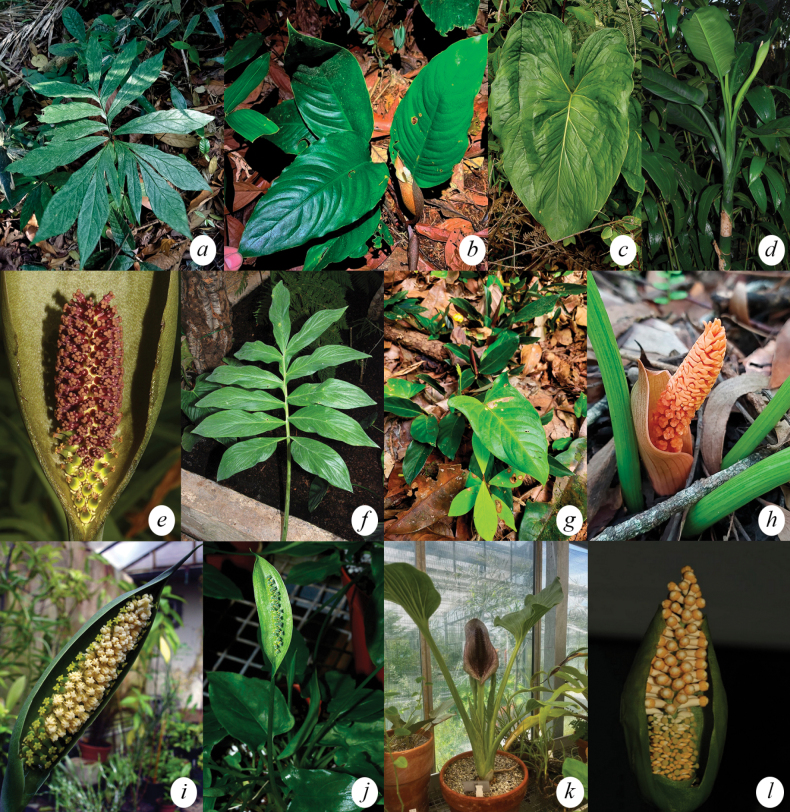
Genera of Spathicarpeae. **a**. *Asterostigma tweedianum* Schott (Picture by Elmar Hentz Júnior); **b**. *Bognera recondita* (Madison) Mayo & Nicolson; **c**. *Croatiella integrifolia* (Madison) E.G. Gonç; **d**. *Dieffenbachia aglaonematifolia* Engl; **e**. *Gorgonidium intermedium* (Bogner) E.G. Gonç; **f**. *Incarum pavonii* (Schott) E.G. Gonç; **g**. *Lorenzia umbrosa* E.G. Gonç. (Picture by Nils Servientis); **h**. *Mangonia tweedieana* Schott (Picture by Luciano Rodrigues Soares); **i**. *Spathantheum orbignyanum* Schott; **j**. *Spathicarpa hastifolia* Hook; **k**. *Synandrospadix vermitoxicus* (Griseb.) Engl (Picture by Elmar Hentz Júnior); **l**. *Taccarum weddelianum* Brongn. Ex Schott (Picture by Elmar Hentz Júnior).

**Figure 3.**
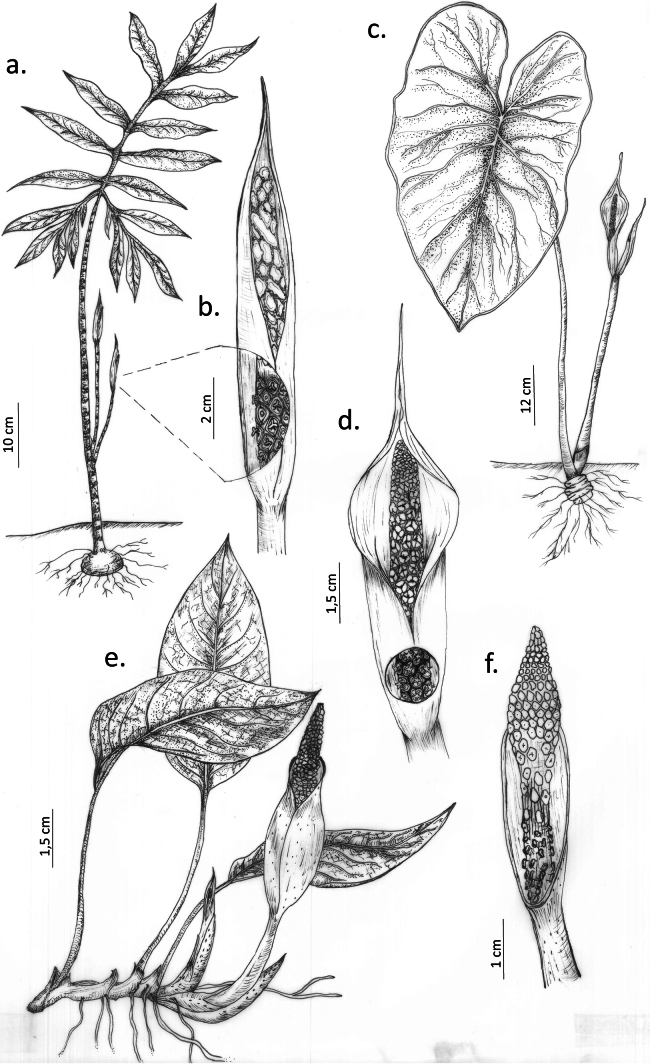
Genera of Spathicarpeae. a. *Asterostigma riedelianum* Schott (Picture by Elmar Hentz Júnior). b. *Bognera recondita* (Madison) Mayo & Nicolson. c. *Croatiella integrifolia* (Madison) E.G. Gonç.

##### Etymology.

The name *Asterostigma* comes from the Greek *astêr* (star) and stigma (stigma), referring to the shape of the stigma of these plants.

##### Species richness and distribution.

*Asterostigma* is represented by 10 species, distributed in Brazil and Argentina (Misiones), within the Atlantic Forest and Cerrado domains, with most species occurring on well-drained soils with an accumulation of leaf litter (Flora e Funga do Brasil 2025).

##### Selected specimens.

Argentina • Ulrich G.E. 5-168 (MO(MO2402793)); Misiones, Colonia General Manuel Belgrano, selva con Arauacaria angustifolia; 20 Oct. 1972. Brazil • Hentz Júnior, E.J. 247 (RB (RB841916)); São Paulo, Atibaia, em cultivo no Horto do Jardim Botânico do Rio de Janeiro; 26 Sep. 2022; fl. • Kassner-Filho A. 5539 (FURB (FURB66284)); Santa Catarina, Itapema, Ilhota; 27°04'6"S, 048°35'54"W; alt. 22m; 30 Sep. 2019; fl. • Hammes, J.K. 266 (UNOP (UNOP11175)); Minas Gerais, Araponga, Parque Estadual da Serra do Brigadeiro; 11 Feb. 2020. • Pereira-Silva G. 6821 (RB (RB448657), CEN); Goiás, Luzuania, Fazenda Alagado; 16°14'07"S, 048°10'41"W; 05 Nov. 2002; fl. • G.S. Siqueira 1293 (CVRD (CVRD15932)); Espírito Santo, Aracruz, Parque Natural Municipal do Aricanga; alt. 295 m; 12 Oct. 2018; fl.

#### Bognera

Taxon classificationPlantaeAlismatalesAraceae

Mayo & Nicolson, Taxon 33: 690 (1984). Type: Bognera recondita (Madison) Mayo & Nicolson ≡ Ulearum reconditum Madison, Aroideana 3(3): 101. (1980)

E42A0AF0-6FA2-58F4-AEB4-F8B1C6094D18

Figs 2b, 3b

##### Diagnosis.

*Bognera* is morphologically similar to *Dieffenbachia* but differs by lacking an aerial stem, by having reticulate venation (vs. pinnate-parallel in *Dieffenbachia*), and by its female flowers lacking staminodes.

##### Etymology.

Named in honor of Dr Josef Bogner (1939–2022), a renowned specialist in Araceae.

##### Species richness and distribution.

*Bognera* is represented by only one species, occurring in Brazil (Acre, Amazonas, and Mato Grosso) and in Peru, in tropical rainforest (“terra firme” forest); terrestrial, creeping over the leaf litter layer on sandy soil (Flora e Funga do Brasil 2025).

##### Selected specimens.

Brazil • M.E. Bleich 14 (INPA (INPA247420)); Mato Grosso, Alta Floresta, Igarapé próximo ao Rio Santa Helena, estrada de acesso à Apiacás; 09°55'10"S, 056°23'01"W, alt. 273 m; 6 May 2011; sterile. • Prance, G.T. 23768 (MO (MO101771837), NY, INPA (INPA211263)); Amazonas, Paumari, Rio Javari, above Attalaia, Forest on terra firme; 14 Oct. 1976; fl.

#### Croatiella

Taxon classificationPlantaeAlismatalesAraceae

E.G.Gonç., Willdenowia 35(2): 323–325, f.3 (2005). Type: Croatiella integrifolia (Madison) E.G. Gonç. ≡ Asterostigma integrifolium Madison Phytologia 35: 101. (1976)

86A581DC-7E67-5BE4-BC71-7F80FAD47BF1

Figs 2c, 3c

##### Diagnosis.

*Croatiella* is characterized by its entire leaves, inflorescence without a sterile apical zone, lobed stigma, and ovary with 5–6 locules.

##### Etymology.

The genus was named in honor of Dr. Thomas Bernard Croat, a specialist in Araceae.

##### Species richness and distribution.

*Croatiella* is represented by only one species, *Croatiella integrifolia*, with a restricted occurrence in western Ecuador, at altitudes of 2,800–3,150 meters (Gonçalves 2005).

##### Selected specimens.

Equador • T. Croat 86435 (MO (MO1772771)); Morona-Santiago, along road from Gualaceo to Limón (Gen. Plaza Guttiérrez), 38.3 km east of Gualaceo (last bridge south of town near city limits); 03°00'57"S, 078°36'14"W; alt. 3,022 m; 10 Aug. 2002, sterile.

#### Dieffenbachia

Taxon classificationPlantaeAlismatalesAraceae

Schott, Wiener Z. Kunst (3): 803, (1829). Type: Dieffenbachia seguine (Jacquin) Schott (“ seguinum”; Arum seguine Jacquin.)

62FB44D3-682E-50AB-BD51-4628B3FE2BCA

Fig. 2d

= Seguinum Rafinesque, Fl. Tell. 3:66 (1837). Type: Seguinum maculatum (G. Lodd) Raf.
= Maguirea A.D.Hawkes, Bull. Torrey Bot. Club 75: 635 (1948). Type: Maguirea spathicarpoides A.D.Hawkes.

##### Diagnosis.

Species of *Dieffenbachia* can be recognized by their epigeous stems, erect to decumbent, usually thick with distinct internodes. It resembles *Bognera*, from which it can be distinguished by presenting a whorl of 4–5 staminodes surrounding the pistillate flowers (vs. absence of staminodes in *Bognera*) and by having pinnate-parallel leaf venation (vs. reticulate in *Bognera*).

##### Etymology.

The genus was named in honor of J. Dieffenbach (1796–1863), chief gardener of the Imperial Palace of Schönbrunn, Austria (Mayo et al. 1997).

##### Species richness and distribution.

*Dieffenbachia* is represented by 57 species, occurring in Mexico, Central America, and much of South America. It has a heliophilous or terrestrial habit, being an important genus in the understory of tropical and subtropical forests, easily noticeable in the landscape due to its large, often variegated leaves (Mayo et al. 1997; Croat and Boyce 2023; Flora e Funga do Brasil 2025).

##### Selected specimens.

Brazil • R.C. Forzza 12557 (RB (RB847591), UPCB, HAMAB, NY); Pará, Almeirim, Estação Ecológica do Jari, Trilha pra Cachoeira da Água Limpa; 00°27'10"S, 052°51'42"W; alt. 300 m; 3 Feb. 2023; fl. • L.C. Ferneda Rocha 268 (UNOP (UNOP72)); Paraná, Foz do Iguaçu, Parque Nacional do Iguaçu, atrás da escola parque; 31 Aug. 2013; fl. Mexico • T. Croat 78695 (WU (WU79378)); Veracruz-Llave, between Catemaco and Montepio; 25 Aug. 1996. Panama • C. Galdames 1222 (B (B100259088)); San Blas, Comarca de San Blas, Sendero Nergan Igar; 02 Jul. 1994. Equador • T. Croat 105781 (MO (MO100867484)); Pastaza, Cabanãs Anzu, gravel road near bridge over Río Anzu and San José de Piatua; 20 Jan. 2015; fr. Peru • R.V. Rocío Rojas 44369 (MO (MO103192671)); Distrito Palcazú, Parque Nacional Yanachaga – Chemillén. Sector Paujil – Quebrada David; 10°23'26"S, 075°17'06"W; alt. 606 m; 20 Oct. 2020; fl.

#### Gearum

Taxon classificationPlantaeAlismatalesAraceae

N.E.Br., J. Bot. 20: 196–197 (1882). Type: Gearum brasiliense N.E.Br.

B8E074EE-0BDC-59AE-BAEA-3A6B038F47FB

##### Diagnosis.

*Gearum* differs from other species of the tribe by having pedate leaves with up to 13 linear leaflets.

##### Etymology.

Derived from the Greek *gê* (earth) and *Arum* (referring to plants of the family Araceae).

##### Species richness and distribution.

*Gearum* is represented by only one species and is endemic to Brazil (Central-West region), occurring in open areas of the Cerrado (Flora e Funga do Brasil 2025).

##### Selected specimens.

Brazil • E.G. Gonçalves 651 (UB (UB0001191)); Tocantins, Arraias, área ao redor do trevo para Paranã e Conceição do Tocantins; 27 Dec. 2000; fl.; fr. • G. Pereira-Silva 11097 (CEN (CEN67362)); Goiás, Teresina de Goias, entrocamento Palmas – Paraná km 2; 12°50'19"S, 047°06'48"W; alt. 400 m; 25 Nov. 2006; fl; fr.

#### Gorgonidium

Taxon classificationPlantaeAlismatalesAraceae

Schott, Ann. Mus. Bot. Lugduno-Batavum 1: 282 (1864). Type: Gorgonidium mirabile Schott.

4EEE07BC-548D-5107-8223-03F3E0274227

Fig. 2e

##### Diagnosis.

It differs from *Synandrospadix* by having filiform to subclavate staminodes, and by the male flowers being free or fused into a synandrium (vs. always completely fused in *Synandrospadix*).

##### Etymology.

Derived from the Greek mythological figure Gorgo, who had snakes as hair, alluding to the filamentous stamens and staminodes of *G. mirabile*.

##### Species richness and distribution.

*Gorgonidium* is represented by eight species, occurring from northern Argentina to Peru, in montane tropical forests between 900 and 3,000 m elevation (Mayo et al. 1997).

##### Selected specimens.

Argentina • T. Croat 68462 (MO (MO374877)); Jujuy, Lagunas de Yala; 26 Nov. 1990. Bolivia • M. Nee 57482 (MO (MO103068180)); Depto. Santa Cruz, Prov. Vallegrande; 18°20'23"S, 064°12'42"W; alt. 1,875 m; 01 Jan. 2011; fl. • J.R.I. Wood 8796 (B (B100275125)); Chuquisaca, Lampacillas (Padilla-Monteagudo); alt. 2,400 m; 19 Nov. 1994. • J.R.I. Wood 22497 (K (K002945040)); Chuquisaca, Zudan~es, c. 10 km of El Rodeo along road to Presto in deep valley; 18°47'81"S, 64°53'41"W; alt. 2,702 m; 18 Nov. 2006; fl.

#### Incarum

Taxon classificationPlantaeAlismatalesAraceae

E.G.Gonç. Willdenowia 35(2): 319–323, f. 1–2 (2005). Type: Incarum pavonii (Schott) E.G.Gonç. ≡ Asterostigma pavonii Schott, Prodromus systematis Aroidearum 339. (1860)

8BC365A0-25D8-5DED-B56B-BF1B8D70F12E

Fig. 2f

##### Diagnosis.

*Incarum* is recognizable by its pinnate leaves, orthotropic ovules, and free, obpyramidal staminodes.

##### Etymology.

The genus name derives from the Inca civilization, as many collections of the genus were made around Inca ruins.

##### Species richness and distribution.

*Incarum* is represented by only one species, *Incarum pavonii*, occurring in the Andes of Bolivia, Ecuador, and Peru, in valleys between mountains at altitudes of 1,200–3,300 m.

##### Selected specimens.

Bolivia • T. Kromer 1742 (MO (MO1498590), LPB); Depto. La Paz, Prov. Nor Yungas, Parque Nacional Cotapata, camino hacia Hormuni Bajo; 16°12'S, 067°52'W; alt. 1,600 m; 23 Nov. 2000; sterile. Peru • J. Lingán 355 (MO (MO101982897)); Distrito Oxapampa. Parque Nacional Yanachaga – Chemillen. Sector San Alberto. Refugio el Cedro; 10°32'S, 075°21'W; alt. 2,400 m; 22 Mar. 2003; fr.

#### Lorenzia

Taxon classificationPlantaeAlismatalesAraceae

E.G.Gonç., Systematic Botany 37 (1): 48–52 (2012). Type: Lorenzia umbrosa E.G.Gonç.

8623DDBA-1A21-5D68-943E-C4AD3E380CB5

Fig. 2g

##### Diagnosis.

*Lorenzia* differs from other species of the tribe by presenting trichomes on the veins and reproductive organs. It is morphologically similar to *Bognera*, from which it differs by having numerous nodes on the stem, always with small cataphylls.

##### Etymology.

The generic name honors Harri Lorenzi, one of the most important botanists in Brazil.

##### Species richness and distribution.

*Lorenzia* is represented by only one species, so far found only in the type locality, Serra do Navio, in Amapá, Brazil, always occurring in very shaded areas (Flora e Funga do Brasil 2025).

##### Selected specimens.

Brazil • E.G. Gonçalves 1224 (UB (Holotype, UB201281)); Amapá, Serra do Navio, 2 km depois de Riozinho, lado esquerdo da BR-210; Jul. 2003; fl.

#### Mangonia

Taxon classificationPlantaeAlismatalesAraceae

Schott, Oesterr. Bot. Wochenbl. 7: 77 (1857). Type: Mangonia tweedieana Schott (“ twedieana”)

2418CAD4-0C0B-556D-A662-E842E8B58357

Fig. 2h

= Felipponia Hicken, Anales Soc. Ci. Argent. 84: 242 (1917), *non Felipponea*Brotherus (1925). Type: Felipponia uruguaya Hicken. Felipponiella Hicken, Darwiniana 2: 30 (1928). Type: Felipponiella uruguaya (Hiken) Bogner.

##### Diagnosis.

Species of *Mangonia* can be recognized by their entire linear to slightly sagittate leaves, free spadix with a terminal appendage of synandrodia anatropous ovules, 2 per locule.

##### Etymology.

No concrete information, but it may derive from the Latin word *mango*, *mangonis* (merchant).

##### Species richness and distribution.

*Mangonia* is represented by two species, occurring in Brazil (Rio Grande do Sul) and Uruguay, in gallery forests with rocky, well-drained soils (Mayo et al. 1997; Croat and Boyce 2023).

##### Selected specimens.

Brazil • R.M. Senna 2921 (HAS (HAS106670)); Rio Grande do Sul, Piratini, Quinto Distrito, Sítio Cambará; 31°35'33.6"S, 053°02'24.7"W; alt. 261 m; 07 Dec. 2023; fl. • E. Barboza 4213 (MBM (MBM393484)); Rio Grande do Sul, São José dos Ausentes, Várzea; 28°29'52"S, 049°47'02"W; alt. 995 m; 27 Sep. 2014; fl. Uruguay • F. Felippone s.n. (MO (MO2514472)); Chucilla de Melo Cerro Largo; 30 Apr. 1917.

#### Spathantheum

Taxon classificationPlantaeAlismatalesAraceae

Schott, Bonplandia 7: 164 (1859). Type: Spathantheum orbignyanum Schott.

E2078B8E-3D6C-5BB7-9101-02479FD60FF7

Fig. 2

= Gamochlamys J.G.Baker, Saunders Refug. Bot. 5: t.346 (1873). Type: Gamochlamys heterandra J.G. Baker.

##### Diagnosis.

It differs from *Spathicarpa* by having a 6–8 locular ovary and pinnate leaves.

##### Etymology.

Derived from the Greek *spathê* (spathe), *anthos* (flower), and *-eum* (a suffix indicating possession), referring to the fusion of the flowers to the spathe.

##### Species richness and distribution.

*Spathantheum* is represented by two species, occurring from northern Argentina to Bolivia, in high-altitude grasslands (Mayo et al. 1997).

##### Selected specimens.

Argentina • A.L. Cabrera 23936 (MBM (MBM30175)); Prov. De Jujuy, Dep. Ledesma, camino a Valle Grande, Abra de Canãs; alt. 1,700m; 07 Nov. 1973; fl. Bolivia • L. Cayola 3620 (MO (MO102669753)); La Paz, Bautista Saavedra, Area Natural de Manejo Integrado Apolobamba, Wayrapata, Kumanita; 15°06'47"S, 068°55'04"W; alt. 2,867 m; 07 May 2010; fr.

#### Spathicarpa

Taxon classificationPlantaeAlismatalesAraceae

Hook. in Bot. Misc. 2: 146 (1831). Type: Spathicarpa hastifolia Hook.

127F2A08-A8B6-5AD1-AEC3-A723870CF828

= [*Spaticarpa* Schott, Oesterr. Bot. Zeitschr. 15: 34 (1965), orth. var.]. (Fig. 2j).
= Aropsis Rojas Acosta, Bull. Acad. Int. Géogr. Bot. 28: 158 (1918). Type: Aropsis palustris Rojas.

##### Diagnosis.

Species of *Spathicarpa* can be recognized by their tuberous stem, spadix completely adnate to the spathe, mixed male and female flowers, and unilocular ovary. It resembles *Spathantheum*, from which it differs by having staminate flowers in two central rows and pistillate flowers in two outer rows (vs. staminate flowers at the apex and pistillate flowers at the base of the spathe in *Spathantheum*).

##### Etymology.

The genus name derives from the Greek *spathê* (spathe) and *karpos* (fruit), referring to these being fused (Mayo et al. 1997).

##### Species richness and distribution.

*Spathicarpa* is represented by four species, occurring in Argentina, Brazil (non-Amazonian), Bolivia, Paraguay, and Uruguay. It has a geophytic habit, occurring from swampy areas in southern Brazil and Paraguay to dry forests with calcareous, well-drained soils (Mayo et al. 1997; Fonsêca et al. 2007; Croat and Boyce 2023; Flora e Funga do Brasil 2025).

##### Selected specimens.

Argentina • W.A. Medina 219 (K (K002976995)); Corrientes, San Martin, Paraje Tres Cerros, Cerro Chico; 29°09'19"S, 056°51'43"W; 26 Oct. 2012; fl. Brazil • D.N.S. Machado 2363 (RFFP (RFFP19878), RB); Rio de Janeiro, Maricá, Pindobas, Pedra do Macaco; 22°55'38"S, 042°53'35"W; alt. 53 m; 21 Nov. 2018; fl. • E.D. Lozano 5102 (MBM (MBM439973)); Paraná, Imbaú, RPPN Samuel Klabin; 24°25'38.7"S, 050°39'33.8"W; 20 Oct. 2021; fl. • R.M. Harley 56592 (HUEFS (HUEFS179369)); Maranhão, Carolina; 20 km da cidade na entrada para Estreito Portal da Chapada; 07°11'13"S, 047°25'23"W; alt. 296m; 19 Jan. 2012; fl.

#### Synandrospadix

Taxon classificationPlantaeAlismatalesAraceae

Engler, Bot. Jahrb. 4: 61 (1883). Type: Synandrospadix vermitoxicus (Grisebach) Engler ≡ Asterostigma vermitoxicum Griseb., Abhandlungen der Königlichen Gesellschaft der Wissenschaften zu Göttingen 19: 247–248. (1874)

6E0F3BEE-F032-577C-8C32-31B492546FAA

Fig. 2k

= Lilloa Spegazzini, Pl. Nov. Crit. Argentina 3: 10 (1897). Type: Lilloa puki Spegazzini.
= *Synandriospadix* Engler, Pflanzenreich 73 (IV.23F): 49 (1920), orth. var.

##### Diagnosis.

It differs from *Gorgonidium* by having entire, slightly cordate leaves (vs. dracontioid leaves in *Gorgonidium*) and completely fused synandria with inconspicuous connectives.

##### Etymology.

The name derives from the Greek *syn* (together), *anêr*, *andros* (man), and *spadix* (spadix), referring to the synandria formed by the fusion of male flowers.

##### Species richness and distribution.

*Synandrospadix* is represented by only one species, occurring from northern Argentina to Peru, in dry subtropical forests (Mayo et al. 1997).

##### Selected specimens.

Argentina • F. Chiarini 647 (CORD); Córdoba, Cruz del Eje, San Marcos Sierras, alrededores de Bariio El Rincón; 27 Nov. 2005. Bolivia • G.A. Parada 2718 (MO (MO100345254)); Santa Cruz, Vallegrande, Camino entre Chañara y Anamal; 18°13'20"S, 064°25'14"W; alt. 1,425 m; 16 Jan. 2011; fr. Paraguay • M. Peña-Chocarro 1905 (BM, MO (MO100242250), FCQ, CTES, G); Presidente Hayes, Laguna Capitán; 18 Oct. 2004; fl.

#### Taccarum

Taxon classificationPlantaeAlismatalesAraceae

Brongniart ex Schott, Oesterr. Bot. Wochenbl. 7: 221 (1857). Type: Taccarum weddellianum Brongniart ex Schott.

2C81D714-DF2D-58E8-980D-4619EA33C876

Fig. 2l

= Lysistigma Schott, Bonplandia 10: 222 (1862). Type: Lysistigma peregrinum Schott.
= Endera Regel, Gartenflora 21: 226 (1872). Type: Endera conophalloidea Regel.

##### Diagnosis.

It is recognized by having tuberous stems, dracontioid (not pedate) leaves, a spadix free or with only part of the female zone adnate to the spathe, elongated synandria with vertical dehiscence, capitate or rounded-lobed stigma, and anatropous ovules.

##### Etymology.

From Malay *taka* (name for *Tacca* in the Taccaceae) and *Arum*, referring to the similarity of the leaf of *Tacca leontopetaloides* to the leaves of this genus.

##### Species richness and distribution.

*Taccarum* is represented by six species, occurring in Argentina (Misiones), Bolivia, Brazil, Paraguay, and Peru, in humid forests and the Cerrado (Croat and Boyce 2023).

##### Selected speciemens.

Bolivia • G.A. Parada 4309 (MO (MO102769506)); Vallegrande, Santa Cruz; 06 May 2012; fr. Brazil • E.D. Lozano 4240 (MBM (MBM428525)); Ceará, Madalena, afluente do Riacho Teotônio; 04°45'22"S, 039°42'22"W; 12 Jun. 2018; fl. • G. Pereira-Silva (CEN (CEN91593)); Maranhão, Carolina, margem esquerda do rio Itapecurpu, próxima à ponte do Melancia; 07°28'36"S, 047°27'40"W; alt. 170 m; 28 Apr. 2008; fr. • M.G. Caxambu 7960 (HCF (HCF23964)); Paraná, Foz do Iguaçu, Parque Nacional do Iguaçu, trilha do Poço Preto; 25°36'22.5"S, 054°25'30.8"W; alt. 255 m; 19 Oct. 2017; sterile. • C.P.C. Azevedo 1 (UFMT (UFMT39235)); Mato Grosso, Nossa Senhora do Livramento, Estância San Raph, BR060 a 35 km de Cuiabá; 15°56.5'S, 057°05.6'W; 22 Oct. 2010; fr.

#### Vivaria

Taxon classificationPlantaeAlismatalesAraceae

O. Cabrera, Tinitana, Cumbicus, Prina & Herrera in PLoS ONE 17 (10) (2022)

18BD1F3A-9907-5AF2-A3A8-478DC826D3E6

##### Type.

*Vivaria calvasensis* O. Cabrera, Tinitana, Cumbicus, Prina & Herrera.

##### Diagnosis.

It differs from other species occurring in Ecuador mainly by presenting fused staminodes, apical synandria present, and sessile synandria (vs. free staminodes, no apical synandria, and pedunculate synandria in *Gorgonidium* and *Incarum*).

##### Etymology.

The generic name honors Francisco Vivar, a recognized professor of botany at the LOJA herbarium.

##### Species richness and distribution.

*Vivaria* is represented by only one species, with known occurrence only at its type locality in southern Ecuador, at altitudes above 1,100 meters (Cabrera et al. 2022).

##### Selected speciemens.

Equador • O. Cabrera 830 (Holotype, HUTPL); Loja province, Calvas canton, 21 km to Bella María; 04°11'59"S, 079°36'56"W; alt. 1,200 m; 30 Jan. 2013; fl.

## Conclusion

Throughout history, different approaches have contributed to the classification of the tribe. Schott (1832, 1856, 1857, 1859a, 1859b, 1860, 1864) emphasized floral morphology. Engler (1878, 1883, 1920) focused on anatomy and vegetative organs, integrating these data into an evolutionary perspective. This line of thought was continued by Bogner and Nicholson (1991), Nicolson (1984), Mayo et al. (1997), who maintained Engler’s view while adding new morphological studies of the taxa. More recently, molecular data were incorporated by authors such as French et al. (1995), Gonçalves et al. (2007), Cusimano et al. (2011), Henríquez et al. (2014), and Haigh et al. (2022), consolidating what is now recognized as Spathicarpeae.

Although distinct, these approaches are not mutually exclusive. On the contrary, they highlight the need for integration of morphology, phylogeny, and taxonomic history, always grounded in the body of knowledge built so far. This combination is essential to understand the diversity of the tribe and to propose a classification system that more accurately reflects its evolutionary relationships.

The bibliographic survey and review of the classification of the tribe Spathicarpeae presented in this study provides a necessary starting point for the development of future phylogenetic studies and taxonomic revisions. Since not all species can be included in molecular analyses, the sampled taxa often serve as representatives of their respective genera. Also, taxonomic revisions generally begin with previously established generic circumscriptions and focus on species delimitation, based on broad sampling of herbarium specimens. In this context, a clear and well-supported definition of these genera is essential for the formulation of consistent evolutionary hypotheses.

## Supplementary Material

XML Treatment for Asterostigma

XML Treatment for Bognera

XML Treatment for Croatiella

XML Treatment for Dieffenbachia

XML Treatment for Gearum

XML Treatment for Gorgonidium

XML Treatment for Incarum

XML Treatment for Lorenzia

XML Treatment for Mangonia

XML Treatment for Spathantheum

XML Treatment for Spathicarpa

XML Treatment for Synandrospadix

XML Treatment for Taccarum

XML Treatment for Vivaria

## Additional information

### Use of AI

No use of AI was reported.

### Author ORCIDs

Elmar J. Hentz Júnior https://orcid.org/0000-0002-8963-4848

Livia G. Temponi https://orcid.org/0000-0003-0449-2703

Marcus A. Nadruz Coelho https://orcid.org/0000-0003-1039-6777

José Fernando A. Baumgratz https://orcid.org/0000-0003-3509-293X

### Data availability

All of the data that support the findings of this study are available in the main text.
